# Individualised patient profile: clinical utility of Flammer syndrome phenotype and general lessons for predictive, preventive and personalised medicine

**DOI:** 10.1007/s13167-018-0127-9

**Published:** 2018-02-22

**Authors:** Olga Golubnitschaja, Josef Flammer

**Affiliations:** 1 Radiological Clinic, Rheinische Friedrich-Wilhelms-Universität Bonn, Sigmund-Freud-Str 25, 53105 Bonn, Germany; 2 Breast Cancer Research Centre, Rheinische Friedrich-Wilhelms-Universität Bonn, Bonn, Germany; 3 Centre for Integrated Oncology, Cologne-Bonn, Rheinische Friedrich-Wilhelms-Universität Bonn, Bonn, Germany; 4 Department of Ophthalmology, University of Basel, Basel, Switzerland

**Keywords:** Predictive preventive personalised medicine, Flammer syndrome, Diagnosis, Cancer, Pregnancy, Risk assessment, Multi-level diagnostics, Recommendations

## Abstract

This case report introduces a female patient, who since her teenager age evidently suffers from Flammer syndrome (FS) as the clearly defined sub-optimal health condition. Further, the patient has experienced collateral pathological conditions which primarily might be linked to the family (genetic) predisposition, but the development of which could be synergistically promoted by the FS-phenotype. The facts are thoroughly analysed and consequent hypotheses are presented, which are indicative for highly desirable predictive diagnostics and targeted preventive measures to be created based on the accurate interpretation of the individualised patient profile. The authors emphasise the great clinical relevance of the FS and field-related research.

## Introduction

Herewith we introduce a patient who evidently suffers from sub-optimal health condition and does worry about highly restricted recognition of the specific signs and symptoms which, however, might be strongly indicative for predictive diagnostic approaches and targeted preventive measures, if a correct interpretation would be made well in time.

## Patient’s history

- General information
  - Female, 55 years old, BMI = 22 kg/m^2^
  - University graduate (biomedical education)
  - Made a successful international scientific career
  - Permanently job occupied since 30 years
  - Taking a good care of her health (regular body exercises, sport vacations, healthy nutrition, well controlled sleep patterns)
- Patient interview
  - Beginning with early childhood, the patient was frequently ill by acute infections being also allergic to some meal products and antibiotics. Her parents tried several approaches to get her health condition more stable such as cryotherapy application which she experienced as horribly stressful.
  - By the family, she has been expected to have the best marks at any level of the school education and in any subject that very early formed her meticulous personality and pronounced tendency to perfectionism.
  - Excellent body shape and high intellectual qualities—both were equally facilitated in her family.
  - In early teenager age, she was the best pupil and the tallest girl in the class. Automatically, everybody expected her to demonstrate extraordinary high sport’s achievements in the school—every time running very quickly as requested led her to a feeling of going to black out but no particular attention was paid to that.
  - The patient does not feel thirsty and drinks too little: first when she feels close to fainting and/or by receiving a headache attack and/or episode of tachyarrhythmia, she recognises potential deficit on a liquid intake.
  - Retrospectively analysing the circumstances, it is getting obvious that her pregnancy was complicated by oligohydramnios which has not been diagnosed timely and contributed to a wrong estimation of the foetus’ size as a very small one. In contrast to the wrong estimates, she gave birth to a son who was 59 cm long and weighed 3.750 kg. Caesarian section was planned in a wrong way for a very small foetus; as the mistake became evident during the operation, more anaesthesia and bigger section was essential to be acutely performed that led to severe complications such as enormous blood lost; the post-surgical recovery has taken several months.
  - As the wound after the Caesarian section was still not completely closed after 4–5 months, for the first time in her life, she was concerned about the delayed or even impaired wound healing which she evidently suffers from, but that has not been diagnosed so far. This observation appeared true, due to strongly prolonged period of time necessary for healing even in case of any small finger cut.
  - Further observations: The patient is extraordinarily touch and pain sensitive; not always but frequently she has dry eyes, nose, mouth and skin, particularly during the winter time; slight nausea is frequent.
  - Specifically in stress situations: she suffers from very cold hands and feet, dry mouth, disturbed movement coordination leading to acute injury which then heals abnormally slowly; her hearing capacity is strongly reduced and she even starts slightly stuttering.
  - During adulthood, her patient records remained quite thin by the treating general practitioner, who considers her health condition “unremarkable”.
- Objective findings
  - Chronic tonsillitis
  - Prolapse of the mitral valve
  - Sporadic cardiac arrhythmia
  - Strong menstrual bleedings related to myomatous disease
  - Ten years ago diagnosed with malignancy (basal cell carcinoma, face) which has been surgically removed; 6 years later diagnosed with the relapse—surgically removed.
  - Signs and symptoms of Flammer syndrome (see the questionnaire filled in below)
- FS signs and symptoms
  FS-related signs and symptoms [1] have been analysed utilising the syndrome-dedicated questionnaire—see Table 1.
- Reproductive history
  - Long menorrhea; regular menstrual cycle since 10 years of age till now
  - Aged 24 years gave birth to one child by caesarean section with complications
  - Thirteen months of breastfeeding
- Lab examination summary

**Table 1:** Specific signs and symptoms are clearly presented in the patient emphasising her strongly pronounced FS-phenotype

Questions	Answers (yes/no)	Comments
• Cold hands and/or feet	Yes	Very frequently even in summer time and particularly in stress situations
• Feel cold	Yes	Very soon if not moving
• Low blood pressure?	Yes	
• Dizziness	Yes	
• Prolonged sleep onset	Yes	Particularly as long as feet are cold and by stress/unsolved problems
• Do not feel thirsty	Yes	Even in hot weather
• Headache/migraine attacks	Yes	Not frequently, but once appears it is getting very strong
• Accompanying symptoms (e.g. visual disturbances)	Yes	Sometimes they appear even before the headache
• Altered reaction towards drugs	Yes	Strongly pronounced
• Altered pain sensitivity	Yes	Extremely touch and pain sensitive
• Strong smell perception	Yes	Extraordinary pronounced
• Slim at 20–30 years of age	Yes	BMI = 19–20
• Tendency towards perfectionism	Yes	Strongly pronounced
• Tinnitus	Yes	Reduced hearing in stress
• Reversible blotches (white or red) on your skin, e.g. in stress situations	Yes	

General clinical biochemical profile in blood is free of any pathological finding. Tumour biomarkers are within the normal range. However, a significantly increased endothelin level by 3.2 pg/ml in blood serum has been detected.

## Family history

- General information
  - All family lines are represented by Caucasians originated from central and Eastern European areas.
  - All members of the family were obligatory literate persons; some of them were speaking several European languages.
  - Members of the family lines were rather asthenic taking care of their body shape and physical aesthetics.
  - Number of children per family corresponded to the average in the region.
  - Socio-economic situation was favourable for all family lines excepting generally difficult periods of wars.
- Disorders in the family
  - No history of genetic diseases.
  - Excepting an accidental death and death, due to acute infectious diseases, the life-duration was sufficiently above average in all the family lines.
  - Grandparents had a history of cardio-vascular diseases and migraine, one cancer case with aggressive metastatic disease.
- Mother
  - Medical doctor who made a successful professional career being job occupied till 70 years of age
  - Extremely stress sensitive person
  - Has not been breastfed after the birth
  - Was very slim in teenager age and adolescence
  - Gave birth to and breastfed two children with 19 years of age difference between them
  - Was taking a good care of her body shape making regular body exercises
  - Suffered from severe headache during regular menstrual periods and experienced an early menstrual onset (11 years of age) and late menopause (60 years of age)
  - Suffered from strong menstrual bleedings related to myomatous disease
  - Had low blood pressure before 40 years of age, however, after that suffered from hypertonic events till the end of her life
  - By the age of 60 years, diagnosed with diabetes mellitus type 2 followed by cardio-vascular disease and severe retinopathy
  - Any medication needed to be individually adapted against the average
  - Died at the age of 77 years from acute thrombosis
- Father
  - Received a complex academic education including medical expertise
  - Made a successful career as administrative manager—job occupied till 75 years of age
  - Extremely stress sensitive person
  - Was very slim in teenager age and adolescence
  - Was taking a good care of his body shape making regular body exercises
  - Had low blood pressure before 40 years of age; afterwards, his blood pressure was normalised
  - Felt less thirsty than average
  - Tinnitus
  - Myocardial infarction at the age of 45 years
  - Diagnosed with the benign prostate adenoma at the age of 60 years
  - Highly sensitive to viral infections
  - Strong vascular dysregulation (cold hands and feet in winter and summer time)
  - Suffered from sleep disorder over several decades of his life
  - Even during hot summer, used heating, due to cold feet, in order to fall asleep in the night
  - Tremor and strongly reduced hearing ability at progressed age
  - Any medication was needed to be individually adapted against the average
  - Died at the age of 88 years from stroke and sudden total collapse of the vascular system

## PPPM-relevant lessons

- What can we learn from the patient’s history presented here?
  - The ancestors, particularly mother and father, had already signs and symptoms of FS. This is very common for such patients. There is little doubt, that we are facing an inherited predisposition, but until now we become just acquainted with the phenotype; the mode of inheritance has not yet been studied.
  - The patient is female. Although FS occurs in both—males and females, the syndrome is clearly more typical for women. This gender difference seems to be less pronounced in Asian people. We assume that the FS-specific hormonal regulation plays a crucial role. This would also explain, why FS symptoms aggravate at puberty and faint after menopause and why oestrogen replacement therapy can boost the symptoms. Central serous chorioretinopathy, a FS-associated condition occurring more often in males, has highest incidence at young age with the highest level of testosterone and can be provoked in females by a testosterone therapy.
  - This patient is a University graduate. This is very typical for FS-affected individuals. The FS occurs clearly more often in academics than in blue collar workers. The causal relationship is still unclear. FS is also more prevalent in subjects with indoor than in subjects with outdoor jobs. We assume a role of the light, although this is not yet proven. FS subjects have often a low level of vitamin D, but light has many additional effects. For example, it boots the production of nitric oxide in the vascular endothelium cells. Therefore, vitamin D dietary supplements do not compensate the UV-deficits.
  - This patient made a brilliant professional carrier. It corresponds well to our experience: FS subjects are very reliable, ambitious and minute in their activities. Also, in this regard, the causal relationship is not yet known.
  - This patient is taking care of her health - both in terms of physical activity and nutrition. Again, this is very characteristic for FS subjects. They are neither lazy nor indifferent but in fact well dedicated and committed. They usually have good knowledge about their own health condition. Patients with a pronounce FS often do intensive sport activities such as bicycling or jogging.
  - She noted an excellent body shape. In general, the lower the BMI, the more pronounced are the FS symptoms. Likewise, the FS signs and symptoms aggravate during fasting periods.
  - This patient experienced the cryotherapy applied with the intention to improve her health condition as horrible. This is not surprising for a number of reasons: the core sign of the FS is an increased response of the vessels to stimuli such as coldness or emotional stress. The coldness obviously induces a pronounced vasoconstriction and this induces pain. Together with an increased pain sensitivity of such subjects, we can easily imagine that the patient enormously suffered from a very uncomfortable condition created by the cryotherapy.
  - The feeling of thirst is clearly reduced in this patient and sometimes she forgets to drink. The low intake of liquid together with the tendency to the low blood pressure makes her feeling close to fainting. This is also characteristic for FS. FS subjects normally have reduced feeling of thirst, most probably because an increased level of endothelin-1 raises production of prostaglandin E-2, which in turn suppresses the feeling of thirst. The main cause of a low blood pressure is most probably a reduced sodium reuptake in the proximal tubuli of the kidneys.
  - The observed delayed wound healing seems to be related to FS, but this relationship needs to be studied. It is feasible, however, that in FS subjects a reduced and unstable blood supply inducing hypoxia and increasing oxidative stress might be unfavourable for the healing. This may also facilitate the growth and metastasis of tumours [2].
- FS Diagnosis

The question is: who should make the FS diagnosis and how [3, 4]? Due to the high specialisation in currently organised medical care, an individual specialist normally does not get such an entire history as thoroughly analysed in our paper. Consequently, patients are often left with the feeling that the different complains represent different diseases or at least different predispositions. This is the situation, where the central role of general practitioners comes into the play. The family doctor normally has a holistic view of the patient. It is already a huge relief for patients, when they realise that the treating physician understands their history and that these complains are not isolated but rather parts of basically one syndrome and that the syndrome is inherited and not a consequence of a misbehaviour. It is a great easement for a patient to hear that their symptoms are comprehensible but not a manifestation of a neurotic personality. The frequent question we receive is: how is it possible that I have all these signs and symptoms, whilst I live such a healthy life? Patients are then often amazed, when they hear that the question “what is healthy” is very individually to reply. Just to give some examples. People in the general population learn permanently that they should reduce salt intake. However, patents with systemic hypotension should rather increase their salt intake. Likewise “being slim”, indeed, is generally healthy, but “being healthy slim” means highly individual BMI usually ranging between 20 and 25 kg/m^2^, and “being too slim” is definitely not optimal at all for being healthy [5, 6]. Unfortunately, the FS is not yet well known amongst physicians; however, it happens frequently, that patients are asking, whether they may be FS-affected, and internet-promoted information distribution regarding FS has significantly contributed to this general trend.

How is the FS diagnosis made? If the signs and symptoms are so clear like in the case of the patient described here, an objective evaluation might even not be necessary. If the symptoms remain doubtful, an objective evaluation is helpful. The physician has to apply accessible approaches. This can be a cold provocation of prolonged vasospasm monitored by capillary-microscopy [7] or a light stimulation on the dynamic retinal vessels analyser monitoring [8], quantification of retinal venous pressure [9], measurements of increased endothelin-1 levels (> 2 pg/ml) in blood serum [10], as well as measurements of the condition-specific molecular patterns in blood [11, 12]. Unfortunately, such tests are still rare and do not belong to conventionally applied medical services.

- What is to do, if FS is diagnosed? PPPM related conclusions and recommendations

It is important to keep in mind that FS is not a disease: the syndrome itself does not need any treatment [13]. However, on the one hand, FS with its well-described signs and symptoms might be very helpful for caregivers regarding the patient’s phenotyping. On the other hand, FS is a sub-optimal health condition which may strongly contribute to the development of severe pathologies, which the FS-affected patients are individually predisposed to [14]. Unfortunately, our current knowledge about the FS-related pathologies is highly limited [15]. However, what is already known is an indication strong enough to be essentially applied for advancing medical services by predictive and preventive measures as well as for personalisation of treatments [16]. Hence, FS is frequently linked to severe eye diseases such as normal-tension glaucoma [17], and particularly aggressive cancer types (metastasing breast cancer) [18], amongst others. A spectrum of severe pathologies, which FS has been linked to, indicates that an individual (e.g. family) predisposition to the concrete pathology may be, further, provoked for its development and progression by the specific FS-phenotype. Contextually, it has been clearly demonstrated that the systemic hypoxia linked to FS generates a particularly “fertile” microenvironment for cancer development and progression into aggressive metastatic disease [18, 19]. Consequently, it is strongly recommended in the case of FS-affected individuals to “zoom” for an individual pathology predisposition, which FS may strongly promote such as the family predisposition to aggressive cancer subtypes [6]. For that, the family history is an essential element in the complex “individualised patient profile” aiming at early and predictive diagnostics.

Contextually, what are the potential PPPM measures to be considered in the future?

1. FS phenotype is highly specific and develops early in life. Consequently, parents should be advised to consult children at the teenager age with family doctors regarding the FS diagnosis in the context of known family disorders and their potential relevance to FS.
2. It makes a very good sense to educate primary caregivers regarding FS, predisposition, diagnostic approach, potentially linked pathologies and targeted preventive measures.
3. Useless and even potentially damaging measures such as cryotherapy should be avoided for the FS-affected individuals, which, in contrast to their positive effects demonstrated in general population, make the FS-affected individuals suffering and could even lead to adverse health effects.
4. Altered drug sensitivity in FS-individuals is a crucial parameter to be carefully considered by caregivers.
5. Pain management is obviously a very specific aspect in medical services provided to the FS-affected individuals that should be carefully considered by personalisation of treatments (e.g. application of anaesthetics in dentistry, surgery, (minimally) invasive diagnostic approaches, amongst others).
6. Reduced thirst feeling typical for the FS-individuals [20] and consequently diminished liquid intake may result in a long-term body dehydration and generation of slightly toxic microenvironment linked to potential complications and even severe pathologies [6]. Some of them are well-acknowledged such as slight nausea mentioned in the patient’s interview (see above), headache/migraine attacks [21] and breast malignancies [22]. Others are just assumed remaining much less investigated such as benign tissue transformation (in case of the described family, this is the prostate adenoma in males and the myomatous disease in females), altered immune response and autoimmune disorders, dry eyes, nose, mouth, cavities, skin and vaginal dryness, liver disorders [23] and potential complications in pregnancy (e.g. oligohydramnious, see the patient description above), amongst others.
7. FS-phenotype is highly relevant to anorexia nervosa (AN), due to the primary vascular dysregulation, low BMI and other signs and symptoms typical for both of them [17]. In turn, AN has been linked to significantly increased risks of compromised immune system, reproductive dysfunction and impaired wound healing, amongst others. The question remains currently unanswered regarding the causality, namely, whether AN might be an extreme case of FS, or FS-phenotype is synergic with other factors (which ones?) collectively promoting the clinical onset of AN [24].
8. Impaired wound healing might be highly relevant for the FS-phenotype with severe consequences such as significantly prolonged post-surgical recovery, chronic wounds and cancer development [2].

The authors strongly emphasise the great clinical relevance of the field-related research to be promoted in accordance with the above listed facts and hypotheses.
